# Multiple Mammarenaviruses Circulating in Angolan Rodents

**DOI:** 10.3390/v13060982

**Published:** 2021-05-25

**Authors:** Jana Těšíková, Jarmila Krásová, Joëlle Goüy de Bellocq

**Affiliations:** 1 Institute of Vertebrate Biology of the Czech Academy of Sciences, 603 65 Brno, Czech Republic; krasova@ivb.cz (J.K.); joellegouy@gmail.com (J.G.B.); 2 Department of Botany and Zoology, Faculty of Science, Masaryk University, 611 37 Brno, Czech Republic; 3 Department of Zoology, Faculty of Science, University of South Bohemia, 370 05 České Budějovice, Czech Republic; 4 Department of Zoology and Fisheries, Faculty of Agrobiology, Food and Natural Resources, Czech University of Life Sciences Prague, 165 00 Prague, Czech Republic

**Keywords:** mammarenaviruses, hantaviruses, Angola, *Mastomys natalensis*, *Micaelamys namaquensis*, *Mus triton*, phylogeny

## Abstract

Rodents are a speciose group of mammals with strong zoonotic potential. Some parts of Africa are still underexplored for the occurrence of rodent-borne pathogens, despite this high potential. Angola is at the convergence of three major biogeographical regions of sub-Saharan Africa, each harbouring a specific rodent community. This rodent-rich area is, therefore, strategic for studying the diversity and evolution of rodent-borne viruses. In this study we examined 290 small mammals, almost all rodents, for the presence of mammarenavirus and hantavirus RNA. While no hantavirus was detected, we found three rodent species positive for distinct mammarenaviruses with a particularly high prevalence in Namaqua rock rats (*Micaelamys namaquensis*). We characterised four complete virus genomes, which showed typical mammarenavirus organisation. Phylogenetic and genetic distance analyses revealed: (i) the presence of a significantly divergent strain of Luna virus in Angolan representatives of the ubiquitous Natal multimammate mouse (*Mastomys natalensis*), (ii) a novel Okahandja-related virus associated with the Angolan lineage of *Micaelamys namaquensis* for which we propose the name Bitu virus (BITV) and (iii) the occurrence of a novel Mobala-like mammarenavirus in the grey-bellied pygmy mouse (*Mus triton*) for which we propose the name Kwanza virus (KWAV). This high virus diversity in a limited host sample size and in a relatively small geographical area supports the idea that Angola is a hotspot for mammarenavirus diversity.

## 1. Introduction

Although arenaviruses (family *Arenaviridae*) have recently been reported in a wide range of vertebrates, e.g., frogfishes, salmon and viperid snakes [1,2,3], they are typically associated with mammals, specifically with muroid rodents (Muroidea). The only exceptions are Tacaribe virus isolated from fruit bats [4] and Alxa virus, which has been described in the northern three-toed jerboa, i.e., in dipodoid rodents (Dipodoidea) [5]. Arenaviruses infecting mammals belong taxonomically to the genus *Mammarenavirus*. Based on antigenic properties, geographical distribution and phylogenetic relationships, members of the genus are further divided into Old World (OW) and New World (NW) groups comprising 19 and 20 distinct mammarenavirus species, respectively [6]. African rodent-borne mammarenaviruses, together with Eurasian representatives, belong to the OW group. They include members capable of causing severe febrile diseases in humans, such as Lassa virus in West Africa [7] and Lujo virus in Zambia [8].

An increasing number of studies have investigated the occurrence of mammarenaviruses in small African mammals, revealing a rich diversity of viruses in rodent hosts. These studies were undertaken in West Africa (e.g., [9] Ghana, [10] Guinea and Nigeria), East Africa (e.g., [11] Ethiopia, [12] Tanzania) and Southern Africa (e.g., [13] South Africa and [14] Namibia). In contrast Central Africa appears understudied. Since the 1983 discovery of Mobala virus (MOBV) in *Praomys* sp. in the Central African Republic [15] only Lymphocytic choriomeningitis virus (LCMV), imported with the invasive house mouse (*Mus musculus*), has been described in Gabon [16]. Even though murid rodents known to be reservoirs of arenaviruses, such as *Mastomys natalensis*, are common in this part of Africa, no additional cases have been reported. This trend is particularly true for Angola, one of the most understudied regions concerning rodent diversity in Africa [17], for which no study has ever assessed rodent-borne viruses.

Angola may be a strategic country to better understand the diversity and evolutionary history of rodent-borne mammarenaviruses, as it is at the crossroads of three major biogeographical regions of sub-Saharan Africa: (1) the Congolian region characterised by moist forests and savannahs; (2) the Zambezian region characterised by woodlands, savannahs and floodplains and (3) the South African region characterised by arid savannahs, dwarf shrublands and desert [18]. This, together with the high diversity of natural habitats, is responsible for a rich rodent community, which in part is formed by a phylogenetic mixture of taxa originating from these three biogeographical regions [17]. From a list of molecular operational taxonomic units (MOTUs)—potential rodent species—given in Krásová et al. [17], several, or their close relatives have been described as reservoirs of mammarenaviruses. Thus, for species having a geographical affinity with the Zambezian region, *Mastomys natalensis* lineage B-VI (sensu Colangelo et al. [19]) is known to be the reservoir of Luna virus (LUAV) in Zambia and Tanzania [20,21] and Mopeia virus (MOPV) in Mozambique and Zimbabwe [22,23]; for species having geographical affinity with the South African region, *Micaelamys namaquensis* is known to harbour Okahandja virus (OKAV) and Mariental virus (MRLV) in Namibia [14]; and finally, for species with geographical affinity with the Congolian region, *Grammomys* sp. is the carrier of Solwezi virus (SOLV) [24]. If we inspect the *Cytochrome b* sequence of the *Grammomys* host associated with the SOLV (GenBank AB972437), it shows 99% identity with *Grammomys surdaster* lineage su7 (sensu Bryja et al. [25]), which is a close relative to the *G. surdaster* lineage su5 recorded by Krásová et al. [17]. Angola, therefore, has the potential to be a hotspot of mammarenavirus diversity.

Another group of rodent-borne viruses, well known as human pathogens, is the family *Hantaviridae*. This group of viruses was only identified in Africa in 2006 [26], though older serological studies suggest it has been circulating on this continent for a longer time [27]. The reservoirs of these viruses are not only limited to rodents but also to bats and soricomorphs (shrews and moles). Although the first hantavirus described on the continent was from the rodent species *Hylomyscus simus* in Guinea, the majority of hantaviruses found in Africa are from bats [28,29,30,31] or shrews [32,33,34,35]. The only two other hantaviruses found in rodents so far are Tigray virus from two endemic species *Stenocephalemys albipes* and *S. zimai* in Ethiopia [11,36], and the globally widespread Seoul virus from the invasive black rat (*Rattus rattus*) in Senegal [37]. Further investigation of a rich rodent community could help gauge if rodents are rare or important reservoir hosts of hantaviruses in Africa.

In this study we investigated if the rich community of Angolan rodents was reflected in the diversity of rodent-borne viruses. For this, we screened the samples collected by Krásová et al. [17] during a trapping survey of rodent diversity in south-western Angola in 2017, for the presence of mammarenaviruses and hantaviruses. We detected three rodent species positive for mammarenaviruses. We then sequenced the full viral genomes before investigating their genetic divergence and phylogenetic relationships with the other OW mammarenaviruses.

## 2. Materials and Methods

### 2.1. Sample Collection

In July 2017, a total of 290 small mammals representing 17 genera, and essentially composed of rodents, were captured across varied biotopes of south-western Angola (Table S1). The trapping protocol (including a description of the nine trapping sites) is described in detail in Krásová et al. [17]. Dried saliva and blood were collected from necropsied animals on Serobuvard^®^ filter papers (Zoopole, Ploufragan, France), and kidney samples in RNAlater stabilisation reagent (Qiagen, Hilden, Germany) which were kept at −80 °C for long-term storage.

### 2.2. Genetic Characterisation of Hosts

Field morphological identification of the rodent species was verified by *Cytochrome b* gene sequencing [38]. Five of the mammarenavirus-positive rodents had already been sequenced in Krásová et al. [17], of which the sequences of *M. triton* were published in a previous evolutionary survey [39]. In this study, we sequenced the *Cytochrome b* gene of the other 19 mammarenavirus-positive individuals as previously described. Host *Cytochrome b* sequences were deposited in GenBank under accession numbers MZ065484-MZ065502 (this study), MW544578-9 and MW544581 [17]. *M. triton* sequences are designated MK011523 and MK011526 [39].

### 2.3. Molecular Screening of Viral RNA

Small pieces of kidney were merged into pools by species and locality, two or a maximum of three individuals per sample, followed by viral RNA extraction with the NucleoSpin RNA Kit (Macherey Nagel, Düren, Germany). Complementary DNA (cDNA) synthesis was performed according to the manufacturer’s instructions using the Maxima^®^ Reverse Transcriptase (Thermo Fisher Scientific, Vilnius, Lithuania) and random hexamers. Samples were then tested for the presence of viruses by PCR assay targeting the *L* gene for arenaviral polymerase [40] using the Phusion Hot Start II DNA Polymerase (Thermo Fisher Scientific, Vilnius, Lithuania) and the *L* gene for hantaviral polymerase [26] using the Multiplex PCR Kit (Qiagen, Hilden, Germany). Mammarenavirus-positive pools were subsequently screened per host to resolve the positive samples. For positive individuals, additional PCRs were performed targeting the *NP* gene for arenaviral nucleoprotein and the *GPC* gene for glycoprotein precursor [41] using Phusion Hot Start II DNA Polymerase. After purification with Exo-CIP clean-up protocol, PCR amplicons were Sanger sequenced at GATC Biotech (Köln, Germany). We used Quantitative Parasitology, version 3.0 [42] to estimate mammarenavirus prevalence with 95% confidence intervals (95% CI) estimated with Sterne‘s exact method [43].

### 2.4. Whole Genome Sequencing and Assembly of Mammarenavirus Genomes

We selected four samples positive for mammarenaviruses, ANG0117 from *M. natalensis*, ANG0052 and ANG0070 from *M. namaquensis* and ANG0206 from *M. triton* for metagenomics. For ANG0070 and ANG0117, we used the RNA previously extracted from kidney tissue. For the two other samples, we extracted RNA from dried blood (ANG0052) and dried saliva (ANG0206) using QIAamp Viral RNA Mini Kit (Qiagen, Hilden, Germany). For ANG0206, cDNA synthesis, library preparation and sequencing followed the procedures described in Goüy de Bellocq et al. [44]. For the three other samples, ribosomal RNA was depleted using the RiboCop rRNA Depletion Kit V1.3 (Lexogen, Vienna, Austria) before RNA library preparation using the Swift RNA Library Kit (Swift Biosciences, Ann Arbor, USA). Reads were quality checked using FastQC [45] and trimmed to remove adapters and low-quality bases using Skewer [46]. De novo assemblies were generated using SPAdes [47]. Assembled contigs were identified using Blobtools [48]. When contigs did not correspond to the full L or S segments, assemblies were completed by iterative mapping using Geneious mapper in Geneious 11.1.5 (Biomatters, Auckland, New Zealand) with the low sensitivity option. This was only necessary in some instances to finalise the assembly of the intergenic regions or the untranslated 3′ and 5′ ends of the segments. The threshold to call nucleotides for the consensus sequences of the L and S segments was set at 75%. Potential N-glycosylation sites were searched for on the NetNGlyc 1.0 server (http://www.cbs.dtu.dk/services/NetNGlyc/ accessed on 10 January 2021).

### 2.5. Genetic Analyses of Mammarenaviruses

Mammarenavirus nucleotide (nt) sequences obtained from the molecular screening were visually inspected in Geneious and manually trimmed for primers or when their quality was low (in the case of the *GPC* gene). Nucleotide viral coding regions of the *GPC*, *NP* and *L* gene were aligned based on translated sequences with a set of homologous sequences of OW mammarenaviruses using MUSCLE [49]. Representatives were selected with an emphasis on the availability of whole-genome sequence, on species accepted by the International Committee on Taxonomy of Viruses (ICTV) and on those with African origin or relevant host species (e.g., we added partial sequences of other viruses harboured by *Nannomys* subgenus-specifically, mammarenaviruses from *Mus minutoides*, *Mus mattheyi*, *Mus setulosus* and *Mus baoulei*). For MRLV described in *Micaelamys namaquensis* in Namibia, the sequence of the L segment available in GenBank (KP867641) shows a fragment of ~870 nt that does not align with other L mammarenavirus sequences and instead shows 98% sequence identity with the MRLV S segment (KM272987). The KP867641 L segment sequence appears to be a chimeric assembly, the first ~4680 nt belonging to the L segment, the next ~870 to the S segment and the last ~1290 nt to the L segment. In the paper describing this virus [14], the authors pointed out that the first sequencing with 454 technology resulted in low coverage and an additional MiSeq sequencing was performed together with Sanger sequencing to complete missing parts of the genome. This virus genome characterisation in several steps may have contributed to the chimeric assembly of the L segment. Thus, only 4209 nt part of the *L* gene corresponding to the portion before the chimeric assembly was used in our phylogenetic analysis.

The phylogeny was reconstructed separately for the three mammarenavirus genes using Bayesian inference (MrBayes 3.2.6.) on the CIPRES web platform [50] using the GTR + I + gamma model of evolution, selected by jModelTest2 under the Bayesian Information Criterion as that best fitting the data [51]. Bayesian posterior probabilities (PP) were used to assess branch support. The Lujo virus was used as an outgroup. Preliminary taxonomic assessment of the detected viruses was examined by the PAirwise Sequence Comparison (PASC) approach [52]. The sequence *p*-distances were further calculated in MEGA X [53].

## 3. Results

### 3.1. Virus Detection and Prevalence

We examined 290 small mammals: nine sengis, one shrew and 280 rodents (two from Bathyergidae, three from Gliridae and 275 from Muridae) for the presence of RNA of mammarenaviruses and hantaviruses. All samples tested negative for hantaviruses. In contrast, 24 samples were found positive for mammarenavirus RNA corresponding to a total prevalence of 8.3% (95% CI: 5.4–12%) (Table S1).

The most frequent host species recorded, *Mastomys natalensis*, represented 32.4% of the sampled animals. In this rodent species, only one individual out of 94 (prevalence 1.1%; 95% CI: 0.1–5.6%) was found to carry a mammarenavirus. BLAST and a preliminary phylogenetic analysis suggested this was a strain of Luna virus (LUAV). We captured this specimen (ANG0117) in Bicuar National Park (Huíla Province) with eight other *M. natalensis*, corresponding to 11.1% (95% CI: 0.6–44.4%) estimated prevalence at the studied locality (Table S1).

Among 75 individuals of *Micaelamys namaquensis*, the second most commonly sampled species, arenavirus RNA was detected in 21 specimens (prevalence 28%; 95% CI: 18.5–39.3%). Specifically, we found 15 positive *M. namaquensis* in Tundavala (local prevalence estimate 40.5%; 95% CI: 25.4–56.8%) and 6 in Bibala (54.5%; 26.5–80%) (Table S1). The prevalence of arenavirus in *M. namaquensis* was not statistically different between the two localities (Fisher’s exact test *p* = 0.5). BLAST and a preliminary phylogenetic analysis of the sequences revealed the presence of a putative novel mammarenavirus, to which we tentatively give the composite name Bitu virus (BITV), after the initial letters of Bibala (Namibe Province) and Tundavala (Huíla Province) localities.

**Figure 1:**
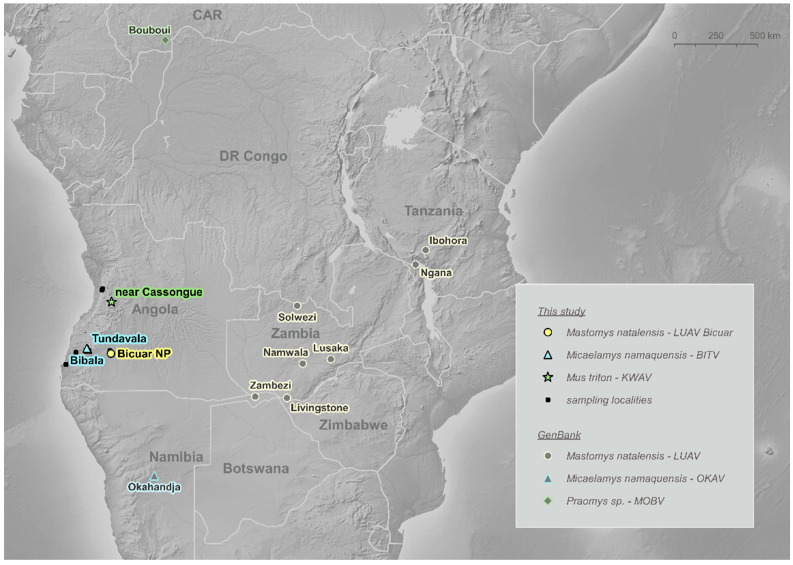
Localities of mammarenavirus-positive rodents from this study and rodents harbouring related mammarenaviruses available in GenBank.

Two out of six individuals of *Mus triton* trapped 20 km SW of Cassongue town (samples ANG0206 and ANG0241; local prevalence 33.33%; 95% CI: 6.3–72.9%) were positive (Table S1). BLAST and a preliminary phylogenetic analysis of the sequences suggested this is a novel mammarenavirus. According to its geographical origin, i.e., an unnamed location near Cassongue, situated in the Kwanza Sul Province, we tentatively named this virus as Kwanza virus (KWAV) (Figure 1).

### 3.2. Characterisation of the Full Viral Genomes

We successfully characterised the complete genomes of four viruses, sample ANG0117 (Luna virus), ANG0052 and ANG0070 (Bitu virus) and ANG0206 (Kwanza virus), apart from six noncoding nt at the 3′ end of the L segment of KWAV. Table 1 summarizes the main characteristics of these genomes and viruses as a whole. Read coverage varied from 46 ± 20 (SD) for the L segment of BITV (ANG0052) to 1674 ± 1339 (SD) for the L segment of KWAV. Each segment for all viruses showed mammarenavirus typical open reading frames (ORFs) separated by the typical stem-loop structures.

The complete L segments are 7187–7296 nt long and contain the two usual *Z* and *L* ORFs. The *Z* ORFs of the four viruses vary between 282–300 nt long and encode 93–99 amino-acid (aa) long zinc finger proteins. The Z proteins contain the regular motifs of OW mammarenaviruses (e.g., conserved RING motif, the conserved N-terminal myristoylation site G2, the late domains PT/SAP, PPXY) except BITV, which shows a shorter C-terminal domain. It is not possible to recognise any known late domains in this virus but, instead, a PTCP sequence that partially mimics the late domain PT/SAP found in most other OW mammarenaviruses. This pattern was already reported for Merino Walk virus [13] and it is also found in OKAV. The *L* ORFs of the viruses vary between 6660 and 6678 nt and encode 2219–2225 aa long RNA-dependent RNA polymerases. In the L protein, the canonical polymerase domains (pre-A, A, B, C, D and E) and the key active site residues of the endonuclease domain NL1 [54] are well conserved for all four viruses.

The complete S segments vary between 3373 and 3476 nt long and contain the two usual *GPC* and *NP* ORFs. The *NP* ORFs of the four viruses, 1689–1710 nt long and encoding 562–569 aa long nucleoproteins, contain aa motifs that resemble other OW mammarenaviruses, including a cytotoxic T-lymphocyte (CTL) epitope GVYMGNL described in LCMV [55]. However, we found one aa change in the CTL epitope of KWAV (GIYMGNL) and a potential antigenic site recorded in the N-terminal portion at position 55–61 [8,56], which has the sequence RKDKRDD for BITV and KWAV, while RKEKRDD for LUAV. The DEDDh motif of the nucleoprotein 3′-5′ exonuclease domain found in other mammarenaviruses is also present. The *GPC* ORFs of our viruses, 1470–1506 nt long and encoding 489–501 aa long glycoprotein precursors, show also usual OW mammarenavirus motifs. The motif at the GP1/GP2 cleavage site of the GPC protein is RRLL for Kwanza, RRLR for Bitu and RRLM for Luna virus. Four to six and three to four N-glycosylation sites can be detected in GP1 and GP2, respectively, in positions analogous to other African mammarenaviruses [57].

#### Special Features of Two Mammarenaviruses

Angolan LUAV shows two characteristics that are unique for this virus. First, the intergenic region of the S segment is large, 164 nt long, compared to other mammarenaviruses for which this region is 60–70 nt-long. A few other mammarenaviruses also have much larger IGRs than average (e.g., Menekre 137 nt-long, Mopeia strain AN20410 123 nt-long) but LUAV has the largest S-segment IGR described so far. The second unique feature is the presence of a motif of 6 nt AAAATT repeated 10 times in the 3′ noncoding region of its L segment. These two features are absent in all other LUAV genomes available in GenBank.

KWAV shows 16 polymorphic nucleotide sites (neither base occurring at a frequency >75%), 15 located in the *L* gene and 1 in the noncoding 5′end of the L segment (see Table S2 for the position of the polymorphic sites). Seven of the 15 polymorphic sites in the *L* gene implied nonsynonymous substitutions occurring at sites showing polymorphism among OW mammarenaviruses. The remaining eight polymorphic sites implied synonymous substitutions (Table S2). This suggests the presence of more than one strain in the saliva of the individual chosen for the sequencing. Polymorphic sites during genome assembly and mapping were previously reported in Gryseels et al. [58] for Gairo virus at 5 nt sites in the S and 1 nt site in the L segment. Because two polymorphic sites occurred at residues that otherwise were conserved in OW mammarenaviruses, the authors suggested these mutations could be due to growth in green monkey-derived Vero E6 cells. In our case, the virus was not previously isolated on cell culture but directly sequenced from dried saliva. No polymorphic sites were detected among the reads in the S segment.

### 3.3. Phylogenetic Characterisation and Molecular Divergence

**Figure 2:**
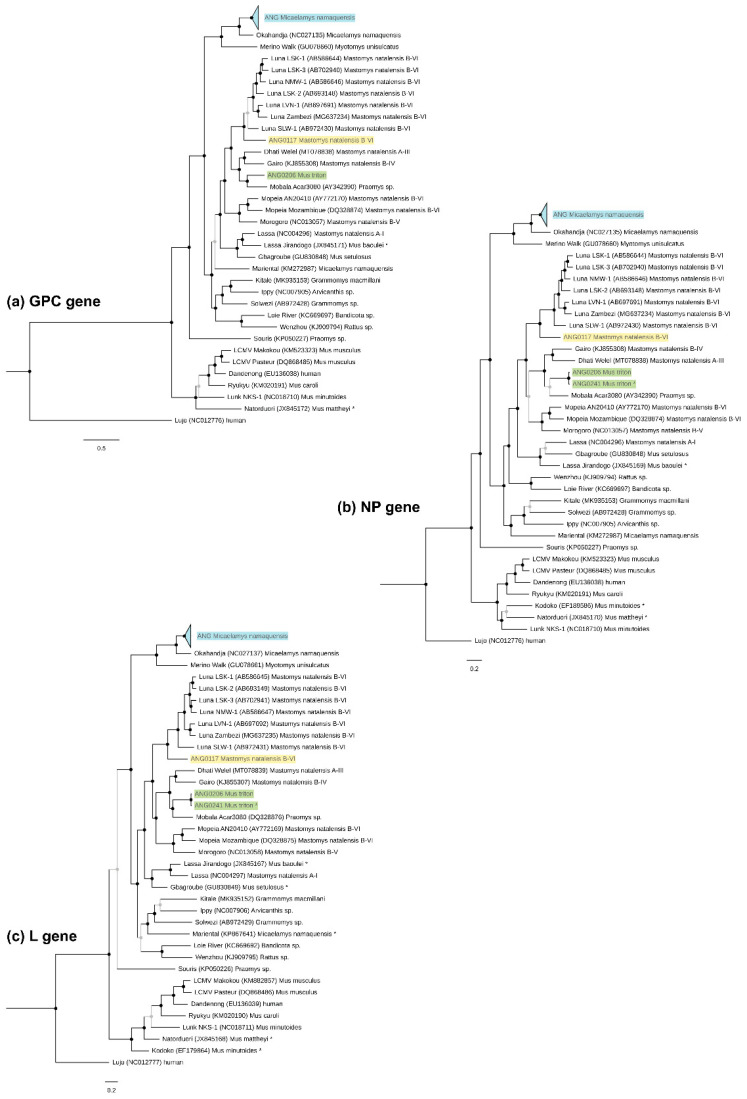
Bayesian phylogenetic trees based on nucleotide sequences of *GPC* (**a**), *NP* (**b**) and *L* (**c**) genes of selected OW mammarenaviruses, with Lujo virus as an outgroup. GenBank numbers of representatives are listed in parentheses. Posterior probability (PP) supports of nodes >0.95 are in black and 0.43 < PP < 0.94 are in light grey. Partial sequences are labeled with asterisks. Viruses described in this study are highlighted by coloured backgrounds: blue for Bitu virus (BITV) from *Micaelamys namaquensis*, yellow for Luna virus (LUAV) strain Bicuar from *Mastomys natalensis* and green for Kwanza virus (KWAV) from *Mus triton*. Part of the BITV phylogeny has been collapsed for clarity (blue triangle) but is provided in Figure S1a.

Based on Bayesian phylogenetic analyses of the *GPC*, *NP* and *L* genes, Angolan LUAV (ANG0117) is basal to all previously identified LUAV strains from Zambia and Namibia, forming a highly supported monophyletic clade (PP = 1, Figure 2). The apparent genetic divergence of Angolan LUAV was identified by the PASC web tool and by evaluating the *p*-distances in MEGA. According to PASC, the closest matches for the complete S segment and L segment sequences were LUAV LSK-2 and LSK-1, with identities of 73.6% and 67.3%, respectively (Table S3a). Thus, less than 80% (S segment) and 76% (L segment), which are the cutoff values established by the ICTV for the assignment of viruses to different species in the genus *Mammarenavirus* [6]. In *p*-distances, none of the other OW mammarenaviruses had >88% identity with Angolan LUAV in the *NP* gene at the aa level (another ICTV criterion), except for LUAV SLW-1 with the pairwise identity of 88.4% (see Table S3b for the summary comparison of the identities).

In addition to the complete genome sequences of ANG0052 and ANG0070 (Table 1), we obtained 11 unique (19 including those identical) sequences of partial *L* gene (339 nt, GenBank MZ065503-13), 12 (19 incl. identical) of the *NP* gene (504 nt, MZ065515-26) and eight (nonidentical) of the *GPC* gene (various lengths between 294 to 744 nt, MZ065528-35). The phylogenetic position, similar for all three genes, grouped the viruses circulating in Angolan *M. namaquensis* with OKAV with high support (PP = 1, Figure 2). The *p*-distance analysis provided sufficiently low identity in aa of the *NP* gene, 85.9% for ANG0052 and 87.2% for ANG0070 with OKAV, to consider BITV as a new species (Table S3c). This was consistent also with PASC analysis: the closest relative for ANG0052 (75.1% in S and 71.6% in L segment) as well as ANG0070 (76.1% and 71.7%) was OKAV (Table S3a). In detail, all phylogenetic trees grouped BITV sequences into one highly supported clade (PP = 1) in which three sequences from Tundavala (partial ANG0017-18 and complete ANG0052) form a sister lineage to all sequences from Bibala (PP = 1), with the remaining Tundavala sequences (including complete ANG0070) forming a third cluster sister to the two previous (PP = 1) (Figure S1a).

Besides the whole genome of KWAV (ANG0206) (Table 1), we obtained *L* (339 nt, GenBank MZ065514) and *NP* (513 nt, MZ065527) gene fragments of sample ANG0241. In the phylogeny, KWAV settled in a cluster of Mobala-like viruses, where it forms a sister lineage of MOBV (found in Bouboui in the Central African Republic), with various support of 0.76 to 1 depending on the gene (Figure 2). The PASC procedure generated the closest hit for KWAV as MOBV: 74.5% and 67.5% nt sequence identity in the S and L segment, respectively (Table S3a). When calculating the distances, KWAV shares with the most related virus (MOBV): 69.7% (*Z* gene), 69.5% (*L*), 75.2% (*GPC*) and 76.7% (*NP*) identities at the nt sequence level and 62.6% (*Z*), 74.3% (*L*), and 88% (*NP*) identities at the aa sequence level. The maximum aa sequence identity of the *GPC* gene (85.7%) shares with Dhati Welel virus found in *Mastomys natalensis* in Ethiopia (Table S3d). Thus, these results suggest KWAV has the genetic properties to be classified as a new species under ICTV criteria.

## 4. Discussion

In this study we detected three different mammarenaviruses in a limited sample size of rodents from a relatively restricted geographical area of Angola (Figure 1), and covering only a subset of the not yet fully characterised Angolan rodent community [17]. Two of these viruses fulfilled the ICTV criteria to be considered as new species. These results are consistent with our hypothesis that this region of Africa is likely a hotspot of mammarenavirus diversity. A second interesting result is the unusually high prevalence of the *M. namaquensis*-borne virus. Finally, one of the new viruses, found in *Mus triton*, clusters in an unexpected part of the OW mammarenavirus phylogenetic tree. In contrast to the mammarenavirus results, no hantavirus was detected in the samples.

The absence of hantavirus detection in our samples supports the idea that rodents are not the most important reservoirs of these viruses in Africa. In fact, only two endemic hantaviruses have been confirmed to circulate in African rodents so far, Sangassou and Tigray [11,26,36], and one imported representative, Seoul, associated with the invasive and commensal black rat [37]. Among the rodent species of Angola listed in Krásová et al. [17], the endemic *Hylomyscus heinrichorum* could be another potential hantavirus reservoir. Indeed, Sangassou virus was not only found in *H. simus* in West Africa, but also more recently in *H. endorobae* in Kenya [31]. Wood mice of the speciose *Hylomyscus* genus are distributed in lowland and montane rainforests of tropical Africa, where they can be locally very abundant. Local abundance is an important criterion that could promote the circulation of viruses [59]. Literature records from the last decade support, instead, a major role of bats and shrews as hantavirus reservoirs on the African continent, but since our virus screening of these groups was limited to a single shrew sample, we are unable to comment further.

The ICTV recommends several criteria should be taken into account when deciding on the validity of a new mammarenavirus species. Among others, its association with a specific host species or group of species, its presence in a defined geographical area and a significant amino acid sequence difference (at least 12% in the nucleoprotein amino acid sequence) from other species in the genus [6]. Two of our viruses, tentatively named BITV and KWAV, fulfil these criteria. Genetic distances (resp. identities) are presented in Table S3. Although BITV shares the same putative host with OKAV from Namibia, the Angolan clade of *M. namaquensis* has recently been defined as a divergent phylogeographic taxon (MOTU 2 in Krásová et al. [17]), potentially a distinct cryptic species from all other *M. namaquensis* inhabiting the South African region, including those from Namibia [60]. For KWAV, this is the first report of mammarenavirus in the rodent species *Mus triton*. Thereby, the association with a specific host species criterion is also met for both new viruses [6].

The relatively high overall prevalence of mammarenaviruses in this sample from Angola, reaching 8.3%, is essentially due to the prevalence of BITV in *M. namaquensis*, which is particularly robust: 28% in this study compared with 1.13% reported for the related OKAV from Namibia [14]. High local prevalences at two sampling sites tens of kilometres apart, Bibala (54.5%) and Tundavala (40.5%), indicate very active BITV infection in the area, probably enhanced by the high population density of the host, which was one of the dominant species in both localities. Interestingly, these two virus-positive localities are separated by the Angolan Escarpment (Figure S1b), a difference in elevation up to 1300 metres, which may be a significant barrier to host contact. Our data show a higher BITV diversity in Tundavala than in Bibala, with the common ancestor of the (Tundavala, Bibala) clade originating from Tundavala (Figure S1a). This is consistent with Bibala being a peripheral isolate which can be explained by its location below the escarpment in the cultivated valley. Tundavala, on the contrary, is situated on the top of the escarpment and is formed by a complex habitat consisting of steep rocky edges, thickets along seasonal streams, miombo woodlands on sands and Afromontane grasslands. This mosaic of different vegetation types likely promotes the genetic structuring in the host and virus compared to the valley where Bibala is located.

Rodents of the subfamily *Murinae* serve as hosts of all known African mammarenaviruses and likewise the genus *Mus*, subgenus *Nannomys*, carries several representatives. For instance, Lunk virus in Zambia, Kodoko in Guinea (both *Mus minutoides*) or Natorduori in Ghana (*Mus mattheyi*) [9,61,62]. These viruses are phylogenetically related to LCMV harboured by the house mouse (*Mus musculus*) worldwide (Figure 2) and which has recently been found also in Gabon (Makokou strain; [16]). In contrast, KWAV clusters in the Mobala-like clade of viruses, typically associated with rodents from the tribe *Praomyini*. This unusual position, pointing to historical host-switching, is not unique. We can see an analogy with other *Nannomys*-borne mammarenaviruses, Gbagroube virus (*Mus setulosus*) and Jirandogo (*Mus baoulei*) from West Africa, which cluster with the LASV complex or even represent a divergent strain of LASV [9,63]. One could argue that these cases are possible spillover infections from members of the *Praomyini* tribe to members of the *Murini* tribe, subgenus *Nannomys*, but this does appear unlikely in three independent cases. Concerning KWAV, we did not find any arenavirus-positive individuals among three other *Nannomys* (four individuals) and two *Mastomys* (18 individuals) subspecies/species captured at the same locality (Table S1). So, it seems that African pygmy mice serve as primary hosts of very divergent lineages of mammarenaviruses.

For the Angolan LUAV, the genetic distance (Table S3a,b) and the unique features of noncoding genomic parts could indicate that it represents a novel species. However, given it is found in the same host as other LUAV strains and that the identity of the *NP* gene with LUAV SLW-1 slightly exceeds the ICTV criterion (88.4% at the aa level), we suggest it is rather a novel LUAV strain. In accordance with this, LUAV strain Bicuar groups with other LUAV strains into a monophyletic clade (Figure 2). The presence of LUAV in the Natal multimammate mouse in Angola, albeit with a low infection rate (1/94), is not surprising, as the virus was originally found in neighbouring Zambia [20] and recently in neighbouring Namibia in the Zambezi region (MG637234-5, GenBank records but no associated paper published yet) (Figure 1). Moreover, *M. natalensis* from these three countries belong taxonomically to the same B-VI mitochondrial clade [17,19], to which LUAV appears to be spatially restricted. The widespread occurrence of LUAV, therefore, correlates with the large range of the host subtaxon B-VI, which inhabits most of Southern Africa [17,19]. It would be interesting to explore whether *M. natalensis* from the bordering Democratic Republic of the Congo, belonging to the A-II clade, host a distinct mammarenavirus.

In summary, we identified Angola as a region of high mammarenavirus diversity, as predicted due to its rich rodent community. Our data support the current assumption about the complex evolutionary history of these viruses with strict host associations coupled with occasional host-switching events. The zoonotic potential of mammarenaviruses should not be underestimated either, as genetic plasticity can be an effective tool to facilitate spillover infection in these viruses [64]. Almost half of mammarenavirus-positive rodents in this study were trapped close to human dwellings; hence, human exposure to infected rodents or contaminated fomites may occur. Although human infections caused by mammarenaviruses are not yet known in this region, future research could investigate the potential epidemiological risk that these viruses may pose to human health. We believe surveillance focusing on rodent fauna and other wild animals as possible zoonotic sources will be worthwhile in this scientifically neglected part of Africa.

## Figures and Tables

**Table 1:** Summary of the different attributes of the four characterised mammarenaviruses.

Virus	Bitu (BITV)	Bitu (BITV)	Luna (LUAV)	Kwanza (KWAV)
Sample	ANG0052	ANG0070	ANG0117	ANG0206
Locality	Tundavala	Tundavala	Bicuar NP	20 km SW of Cassongue
Host species	*Micaelamys* *namaquensis*	*Micaelamys* *namaquensis*	*Mastomys* *natalensis*	*Mus* *triton*
GenBank # Host Cyt b	MZ065491	MW544581	MZ065496	MK011523
GenBank # L segment	MZ065536	MZ065538	MZ065542	MZ065540
GenBank # S segment	MZ065537	MZ065539	MZ065543	MZ065541
Coverage L segment	46 ± 20	98 ± 41	319 ± 113	1674 ± 1339
Coverage S segment	51 ± 24	63 ± 30	761 ± 263	1660 ± 1240
Length L segment (nt)	7187	7223	7296	7222
Length L ORF (aa)	2225	2225	2219	2220
Length Z ORF (aa)	93	93	98	99
Length S segment (nt)	3374	3373	3476	3421
Length GPC ORF (aa)	501	501	491	489
Length NP ORF (aa)	562	562	569	569
Late domains in Z	PTCP-none	PTCP-none	PTAP-PPPY	PTAP-PPAY
CTL epitope in NP *	GVYMGNL	GVYMGNL	GVYMGNL	GIYMGNL
Antigenic site in NP **	RKDKRDD	RKDKRDD	RKEKRDD	RKDKRDD
GP1/GP2 cleavage site	RRLR	RRLR	RRLM	RRLL
N-glycosylation GP1	6	6	4	5
N-glycosylation GP2	3	4	3	3

* cytotoxic T-lymphocyte (CTL) epitope “GVYMGNL” listed in the nucleoprotein of LCMV. ** antigenic site “RKSKRND” reported in the N-terminal portion of LASV nucleoprotein.

## Data Availability

The data presented in this study are available on request from the corresponding author.

## Supplementary Materials

The following are available online at https://www.mdpi.com/article/10.3390/v13060982/s1, Figure S1: (a) Detailed view at the uncollapsed part of the Bitu virus (BITV) phylogeny and (b) Angolan Escarpment (photo N. Baptista, in Tundavala). Table S1: species list. Table S2: polymorphic sites of KWAV. Table S3: sequence identities (a) by PASC, (b) of ANG0117, (c) of ANG0052/ANG0070 and (d) of ANG0206.
