# Supplementary material for: Multiple Mammarenaviruses Circulating in Angolan Rodents
Source: Viruses. 2021 May 25;13(6):982. doi: 10.3390/v13060982 (PMC8227972; doi:10.3390/v13060982)
Supplement: Supplementary file 1 [file viruses-13-00982-s001.zip › Table S1 Species list.pdf]

Supplementary Table 1 PCR detection of mammarenavirus cDNA in Angolan small mammals

Family	Genus	Species	Capture Site	Longitude	Latitude	Altitude	Arenavirus Positive	Individuals Tested	Arenavirus Prevalence in % [95% CI]
Bathyergidae	*Fukomys*		Hombe	14.77175	-14.90245	1261	0	1	-
			Tundavala	13.40352	-14.81098	2283	0	1	-
Gliridae	*Graphiurus*		Hombe	14.77175	-14.90245	1261	0	2	-
			Tundavala	13.40352	-14.81098	2283	0	1	-
Macroscelididae	*Elephantulus*		Caraculo	12.651958	-15.027038	610	0	3	-
			Tundavala	13.40352	-14.81098	2283	0	6	-
Muridae	*Aethomys*		Kumbira	14.29293	-11.15439	881	0	14	-
	Dendromus		20 km SW of Cassongue	14.87526	-11.91103	1752	0	2	-
	Gerbilliscus		Arco oasis	12.064888	-15.768228	43	0	5	-
			Bicuar National Park	14.83968	-15.10135	1242	0	6	-
			Hombe	14.77175	-14.90245	1261	0	1	-
			Tundavala	13.40352	-14.81098	2283	0	13	-
	Hylomyscus		20 km SW of Cassongue	14.87526	-11.91103	1752	0	3	-
	Lophuromys		Kumbira	14.29293	-11.15439	881	0	12	-
			20 km SW of Cassongue	14.87526	-11.91103	1752	0	2	-
	Mastomys	*angolensis*	20 km SW of Cassongue	14.87526	-11.91103	1752	0	17	-
	Mastomys	*natalensis*	Arco oasis	12.064888	-15.768228	43	0	1	-
			Bibala	13.35047	-14.75923	912	0	7	-
			Bicuar National Park	14.83968	-15.10135	1242	**1**	9	**11.1** [0.6-44.4]
			Conda	14.31559	-11.101137	925	0	12	-
			Hombe	14.77175	-14.90245	1261	0	30	-
			20 km SW of Cassongue	14.87526	-11.91103	1752	0	1	-
			Tundavala	13.40352	-14.81098	2283	0	34	-
	Micaelamys	*namaquensis*	Arco oasis	12.064888	-15.768228	43	0	10	-
			Bibala	13.35047	-14.75923	912	**6**	11	**54.5** [26.5-80]
			Caraculo	12.651958	-15.027038	610	0	3	-
			20 km SW of Cassongue	14.87526	-11.91103	1752	0	14	-
			Tundavala	13.40352	-14.81098	2283	**15**	37	**40.5** [25.4-56.8]
	Mus		ISCED Lubango	13.48478	-14.91724	1807	0	1	-
			Kumbira	14.29293	-11.15439	881	0	7	-
			20 km SW of Cassongue*	14.87526	-11.91103	1752	0	4	-
	Mus	*triton*	20 km SW of Cassongue	14.87526	-11.91103	1752	**2**	6	**33.33** [6.3-72.9]
	Petromyscus		Caraculo	12.651958	-15.027038	610	0	2	-
	Rattus		Conda	14.31559	-11.101137	925	0	1	-
			Kumbira	14.29293	-11.15439	881	0	4	-
	Rhabdomys		Tundavala	13.40352	-14.81098	2283	0	3	-
	Steatomys		Tundavala	13.40352	-14.81098	2283	0	1	-
	Thallomys		Caraculo	12.651958	-15.027038	610	0	1	-
			Hombe	14.77175	-14.90245	1261	0	1	-
Soricidae	*Crocidura*		20 km SW of Cassongue	14.87526	-11.91103	1752	0	1	-
Total							**24**	290	**8.3** [5.4-12]

* 2 *Mus cf. kasaicus* , 1 *Mus minutoides* (sensu Bryja *et al.* [65]; *cyt b* analysis) and 1 *Nannomys sp. indet.* (assumption based on morphology)
