# Supplementary material for: Multiple Mammarenaviruses Circulating in Angolan Rodents
Source: Viruses. 2021 May 25;13(6):982. doi: 10.3390/v13060982 (PMC8227972; doi:10.3390/v13060982)
Supplement: Supplementary file 1 [file viruses-13-00982-s001.zip › Table S2 Polymorphic sites of KWAV.pdf]

Supplementary Table 2 Polymorphic sites in the L segment of ANG0206 (KWAV)

Position in the L segment	Location in the L segment	Change	Codon Change	Coverage	Protein Effect	Amino Acid* Change	Variant Frequency
91	5' end	T → C	-	3548	-	-	34.1%
673	L gene	T → C	ACC → GCC	1399	Substitution	T → A	46%
790	L gene	T → C	AGT → GGT	1487	Substitution	S → G	39.5%
890	L gene	T → C	GAA → GAG	1442	None	-	43.1%
1297	L gene	T → C	ATT → GTT	2289	Substitution	I → V	38.4%
1322-1323	L gene	GG → AC	ACC → AGT	2097-2108	Substitution	T → S	33.3% - 33.4%
2160	L gene	A → G	GTT → GCT	2104	Substitution	V → A	35.9%
2393	L gene	G → A	TCC → TCT	2467	None	-	33.8%
3971	L gene	G → A	CAC → CAT	1758	None	-	28.4%
4004	L gene	G → A	TGC → TGT	2457	None	-	37%
4121	L gene	C → T	CAG → CAA	5400	None	-	37.5%
4371	L gene	T → C	AAA → AGA	3751	Substitution	K → R	36.6%
5687	L gene	T → C	AAA → AAG	387	None	-	39%
6023	L gene	C → T	GTG → GTA	1604	None	-	41.6%
6698	L gene	G → A	GCC → GCT	806	None	-	36.4%

* A = Alanine, G = Glycine, I = Isoleucine, K = Lysine, R = Arginine, S = Serine, T = Threonine, V = Valine
