# Supplementary material for: Multiple Mammarenaviruses Circulating in Angolan Rodents
Source: Viruses. 2021 May 25;13(6):982. doi: 10.3390/v13060982 (PMC8227972; doi:10.3390/v13060982)
Supplement: Supplementary file 1 [file viruses-13-00982-s001.zip › Table S3 Sequence identities.pdf]

Supplementary Table 3a PASC nucleotide sequence identities comparison of Angolan mammarenaviruses within Arenaviridae

Sample	nt sequence identity S segment (in %)	The closest hit species - Genbank #	nt sequence identity L segment (in %)	The closest hit species - Genbank #
ANG0052	75.1	Okahandja - NC027135	71.6	Okahandja - NC027137
ANG0070	76.1	Okahandja - NC027135	71.7	Okahandja - NC027137
ANG0117	73.6	Luna LSK2 - AB693148	67.3	Luna LSK1 - NC016153
ANG0206	74.5	Mobala - NC007903	67.5	Mobala - NC007904

Supplementary Table 3b Nucleotide sequence and amino acid identity comparison between ANG0117 and OW mammarenaviruses

		GPC gene		NP gene			L gene		Z gene	
Virus	Genbank # S segment	nt (%)	aa (%)	nt (%)	aa (%)	Genbank # L segment	nt (%)	aa (%)	nt (%)	aa (%)
Luna SLW-1	AB972430	76.3	88.4	73.7	**88.4**	AB972431	67.3	71.6	64.3	71.4
Luna LSK-2	AB693148	75.2	89.2	75.3	87.6	AB693149	68.3	72.4	65.3	**72.4**
Luna Zambezi	MG637234	75.6	87.4	74.1	87.2	MG637235	67.0	70.9	67.0	70.4
Luna LSK-1	AB586644	75.4	**89.4**	74.0	87.2	AB586645	68.8	72.5	66.0	**72.4**
Luna LSK-3	AB702940	73.9	88.6	73.9	87.0	AB702941	68.7	72.5	64.6	68.4
Luna LVN-1	AB697691	75.7	88.4	74.8	87.0	AB697692	67.8	72.0	66.7	70.4
Luna NMW-1	AB586646	75.2	**89.4**	74.3	86.6	AB586647	68.7	**72.8**	64.3	69.4
Mopeia	AY772170	68.5	76.5	71.9	81.7	AY772169	62.3	62.8	61.3	63.3
Morogoro	NC013057	68.9	79.6	70.3	81.0	NC013058	61.9	62.3	56.6	59.2
Dhati Welel	MT078838	70.8	80.3	70.5	78.6	MT078839	62.3	62.7	66.0	66.7
Mobala	AY342390	69.5	80.8	69.7	77.8	DQ328876	63.1	63.2	60.8	63.5
Gairo	KJ855308	71.3	79.3	69.9	77.3	KJ855307	62.7	63.2	61.0	59.1
Lassa	NC004296	67.2	75.4	69.0	75.5	NC004297	59.3	57.3	58.4	58.3
Wenzhou	KJ909794	64.0	69.9	67.5	75.0	KJ909795	57.3	54.0	60.7	61.3
Loie River	KC669697	64.6	70.1	65.3	73.0	KC669692	57.5	54.6	62.1	63.2
Ippy	NC007905	67.6	73.0	65.9	72.5	NC007906	57.8	55.3	58.9	58.5
Solwezi	AB972428	67.0	74.1	66.1	71.0	AB972429	57.4	54.5	62.5	62.8
Kitale	MK935153	66.7	72.4	65.7	70.4	MK935152	57.7	54.7	57.8	55.9
Merino Walk	GU078660	63.1	68.5	64.9	68.4	GU078661	56.8	53.1	59.5	56.3
Okahandja	NC027135	63.5	67.8	65.1	67.3	NC027137	55.9	51.3	55.3	56.3
Dandenong	EU136038	59.8	57.5	62.8	65.7	EU136039	53.5	48.3	57.4	51.7
LCMV	DQ868485	57.5	56.7	62.1	65.7	DQ868486	54.3	48.3	57.4	52.8
Ryukyu	KM020191	57.9	56.9	61.5	65.5	KM020190	53.2	47.7	54.4	50.6
Lunk	NC018710	58.3	57.1	61.4	63.7	NC018711	52.9	47.9	50.0	48.4
Lujo	NC012776	51.5	42.6	60.2	59.1	NC012777	51.9	45.0	52.3	44.8

Supplementary Table 3c Nucleotide sequence and amino acid identity comparison between ANG0052 / ANG0070 and OW mammarenaviruses

		GPC gene		NP gene			L gene		Z gene	
Virus	Genbank # S segment	nt (%)	aa (%)	nt (%)	aa (%)	Genbank # L segment	nt (%)	aa (%)	nt (%)	aa (%)
in common		80.0	89.8	82.7	92.0		80.4	88.1	82.3	84.9
Okahandja	NC027135	74.2 / 75.4	**84.5** / **84.2**	76.2 / 76.7	**85.9** / **87.2**	NC027137	73.0 / 73.1	**79.8** / **79.3**	72.4 / 71.7	**78.3** / **76.1**
Merino Walk	GU078660	66.2 / 66.8	75.4 / 76.1	70.6 / 71.3	79.7 / 79.9	GU078661	63.4 / 63.0	63.7 / 63.6	59.6 / 59.3	61.8 / 62.9
Wenzhou	KJ909794	61.6 / 61.3	62.2 / 64.2	66.5 / 66.2	69.8 / 70.7	KJ909795	55.1 / 55.7	52.0 / 51.8	59.0 / 56.6	55.1 / 53.8
Ippy	NC007905	64.7 / 64.2	70.6 / 73.3	65.0 / 64.1	69.0 / 69.2	NC007906	56.4	52.2 / 52.4	56.0 / 56.7	54.8
Solwezi	AB972428	65.3 / 64.5	69.5 / 71.6	64.8 / 64.2	68.6	AB972429	56.1 / 56.2	53.3 / 52.7	57.3 / 56.6	54.3 / 53.3
Mopeia	AY772170	64.3 / 64.1	68.0 / 67.9	63.4 / 63.2	68.5 / 69.0	AY772169	57.3 / 56.4	52.7 / 52.6	56.9 / 54.3	50.5 / 52.7
Kitale	MK935153	62.8 / 63.8	68.5 / 70.2	65.6 / 65.7	68.4 / 69.3	MK935152	55.6 / 56.0	53.2 / 53.1	51.6 / 49.5	47.8 / 45.6
Loie River	KC669697	63.7 / 63.5	67.1 / 67.9	65.4 / 64.0	68.0 / 68.9	KC669692	56.7 / 56.6	53.7 / 53.3	59.4 / 61.2	56.0 / 58.2
Lassa	NC004296	65.7 / 66.4	69.3 / 69.0	63.9 / 63.8	67.8 / 68.3	NC004297	56.6	52.2 / 51.9	61.4 / 56.8	63.2 / 60.9
Morogoro	NC013057	63.7 / 65.9	67.2 / 68.7	64.1 / 64.3	67.8 / 68.3	NC013058	56.6 / 55.9	52.7 / 52.6	52.2 / 52.9	50.5
Dhati Welel	MT078838	63.4 / 65.6	68.0 / 69.1	64.7 / 64.6	67.8 / 68.1	MT078839	56.9 / 56.2	53.0 / 52.4	60.8 / 59.0	58.9 / 60.0
Gairo	KJ855308	64.2 / 65.3	67.6 / 69.0	64.4 / 63.5	67.6 / 66.8	KJ855307	57.2 / 57.5	52.9 / 53.1	54.6 / 57.1	48.9 / 51.1
Luna	AB586644	64.9 / 65.4	68.0 / 68.4	63.1 / 63.9	67.4 / 68.1	AB586645	56.2 / 56.7	51.8 / 52.2	60.3 / 56.2	55.7 / 56.8
Dandenong	EU136038	59.1 / 59.2	57.0 / 57.7	63.5 / 62.5	66.2 / 66.7	EU136039	52.2	46.2	58.7 / 54.9	55.2
Mobala	AY342390	63.5 / 64.1	67.1 / 68.6	64.1 / 64.3	66.1 / 67.0	DQ328876	57.2 / 57.1	52.9 / 53.0	58.2 / 60.1	60.0 / 58.9
Ryukyu	KM020191	58.3 / 58.1	54.5 / 56.0	63.4 / 62.2	65.6	KM020190	52.6 / 52.9	46.4 / 46.7	58.0 / 54.5	57.5
LCMV	DQ868485	59.1 / 59.3	56.2 / 56.9	63.3 / 62.2	65.3 / 65.6	DQ868486	52.3	46.3 / 47.1	57.2 / 54.2	51.7
Lunk	NC018710	58.7 / 58.0	55.7 / 55.9	63.1 / 62.5	64.7 / 66.0	NC018711	51.5 / 52.0	46.6 / 46.5	54.8 / 51.5	49.4
Lujo	NC012776	51.0 / 50.4	42.8 / 42.6	60.9 / 61.3	59.9 / 59.4	NC012777	51.8 / 51.1	45.1	52.0 / 48.4	40.2 / 39.1

Supplementary Table 3d Nucleotide sequence and amino acid identity comparison between ANG0206 and OW mammarenaviruses

		**GPC gene**		**NP gene**			**L gene**		**Z gene**	
Virus	Genbank # S segment	nt (%)	aa (%)	nt (%)	aa (%)	Genbank # L segment	nt (%)	aa (%)	nt (%)	aa (%)
Mobala	AY342390	75.4	85.1	76.7	**88.0**	DQ328876	69.5	**74.3**	69.7	**62.6**
Gairo	KJ855308	73.0	83.0	73.0	84.9	KJ855307	68.4	72.2	64.3	61.5
Dhati Welel	MT078838	74.6	**85.7**	72.9	84.3	MT078839	68.5	71.4	61.2	61.6
Mopeia	AY772170	68.5	78.1	71.4	78.6	AY772169	64.0	64.1	60.6	56.1
Luna	AB586644	71.7	80.4	70.8	78.7	AB586645	62.7	63.3	62.9	57.7
Morogoro	NC013057	70.2	79.4	71.8	77.9	NC013058	62.0	62.5	56.2	55.1
Lassa	NC004296	68.4	77.8	68.8	73.4	NC004297	60.0	58.7	62.9	58.3
Wenzhou	KJ909794	65.5	72.1	66.6	71.4	KJ909795	57.6	55.0	60.5	59.0
Loie River	KC669697	66.7	73.5	65.9	70.2	KC669692	57.2	54.9	58.2	61.1
Solwezi	AB972428	67.9	74.8	65.0	68.9	AB972429	57.9	55.3	64.6	62.5
Ippy	NC007905	65.7	72.3	64.2	68.6	NC007906	58.1	56.0	59.9	58.8
Kitale	MK935153	66.5	71.7	64.1	68.3	MK935152	57.0	55.3	59.8	59.4
Merino Walk	GU078660	64.8	69.7	63.8	65.9	GU078661	57.0	52.9	55.9	52.8
Okahandja	NC027135	64.2	67.9	63.6	65.7	NC027137	56.2	52.1	57.4	58.4
Dandenong	EU136038	59.1	59.2	61.9	63.5	EU136039	53.4	47.7	57.1	50.0
Lunk	NC018710	59.0	57.1	61.3	63.3	NC018711	53.1	48.3	55.0	47.3
LCMV	DQ868485	59.1	58.6	61.9	62.9	DQ868486	54.1	47.9	55.3	47.8
Ryukyu	KM020191	60.1	58.8	59.8	61.9	KM020190	52.9	47.2	54.2	48.9
Lujo	NC012776	51.0	44.2	60.7	60.1	NC012777	52.1	45.5	49.6	40.4
