# Supplementary figures and images for: Multiple Mammarenaviruses Circulating in Angolan Rodents
Source: Viruses. 2021 May 25;13(6):982. doi: 10.3390/v13060982 (PMC8227972; doi:10.3390/v13060982)

a) detailed BITV phylogeny

GPC gene

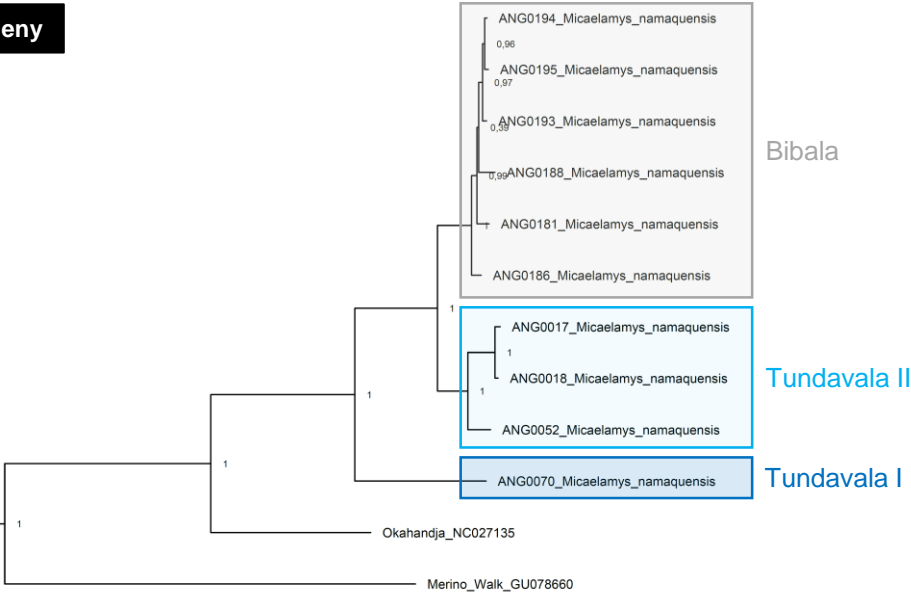

0.5

NP gene

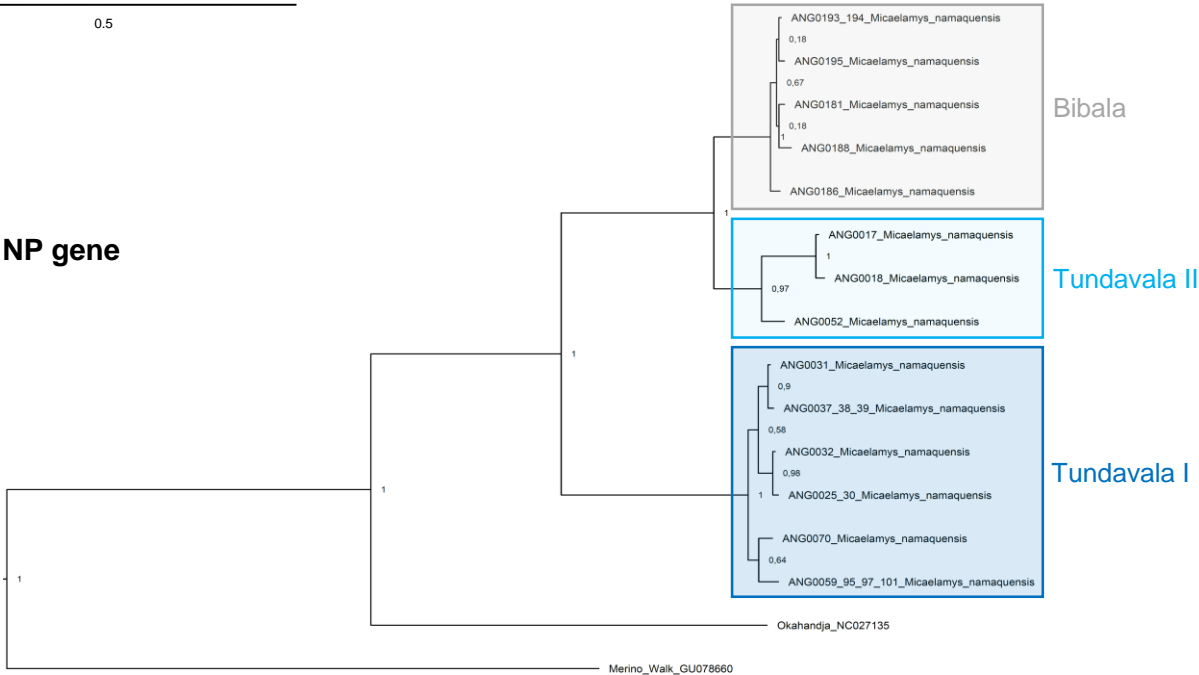

0.2

L gene

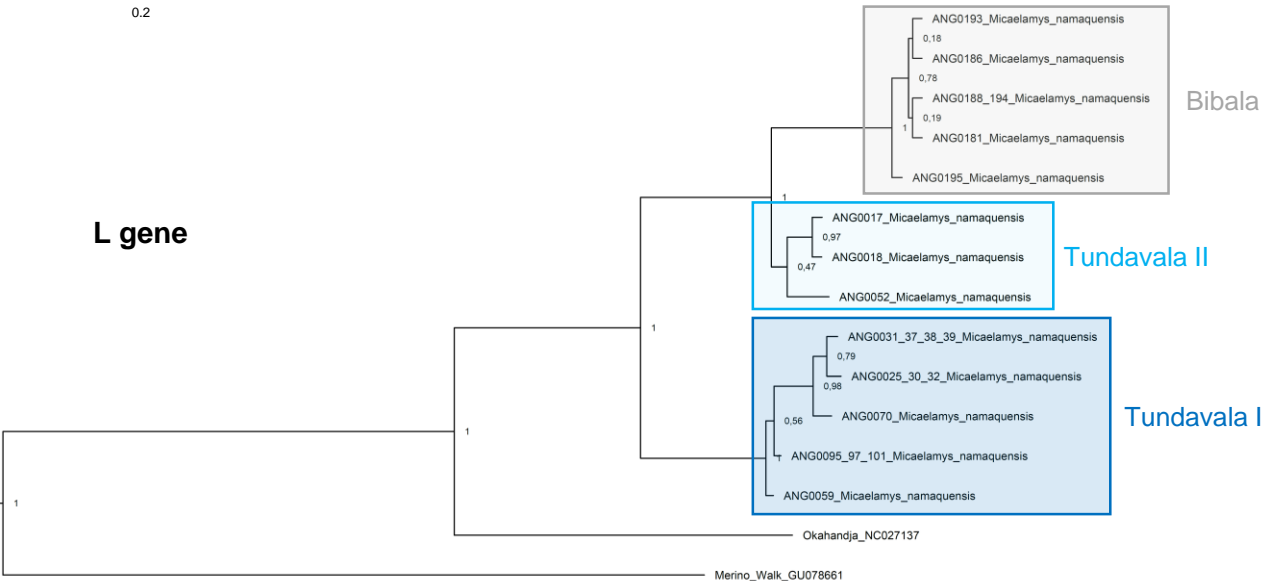

0.2

## b) Angolan Escarpment

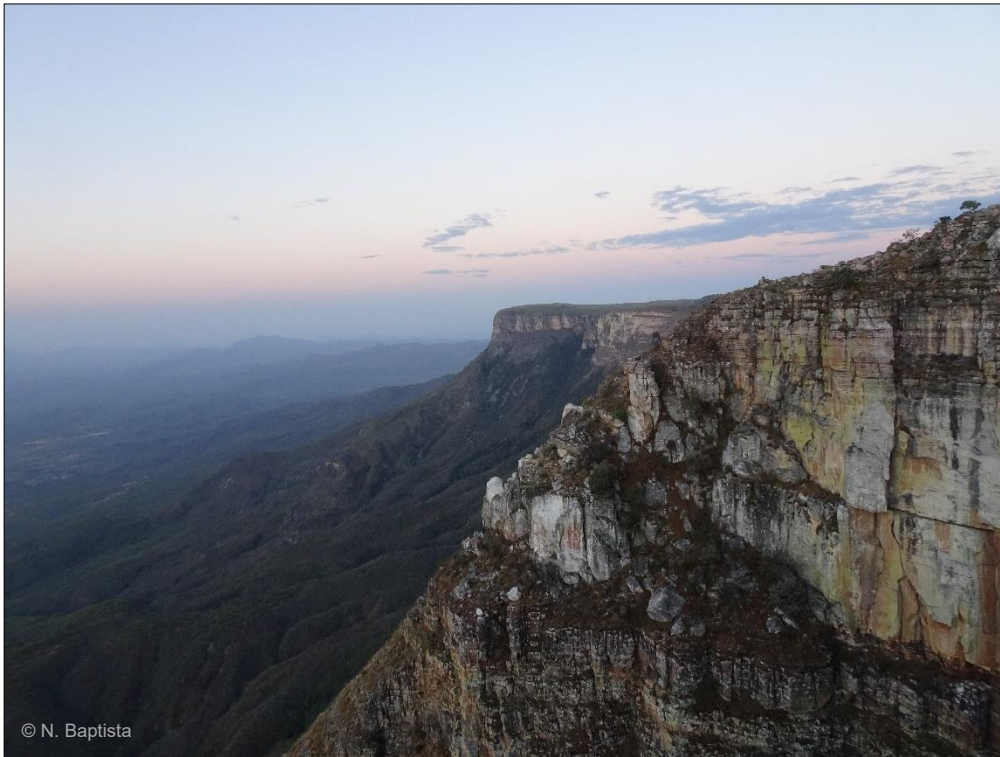

© N. Baptista

Supplement: Supplementary file 1 [file viruses-13-00982-s001.zip › Figure S1 BITV detailed.pdf]
